# Epidemiology and outcome of severe pneumococcal pneumonia admitted to intensive care unit: a multicenter study

**DOI:** 10.1186/cc11471

**Published:** 2012-08-15

**Authors:** Nicolas Mongardon, Adeline Max, Adrien Bouglé, Frédéric Pène, Virginie Lemiale, Julien Charpentier, Alain Cariou, Jean-Daniel Chiche, Jean-Pierre Bedos, Jean-Paul Mira

**Affiliations:** 1 Medical Intensive Care Unit, Cochin Hospital, Groupe Hospitalier Universitaire Cochin-Broca-Hôtel-Dieu, Assistance Publique des Hôpitaux de Paris, 27 rue du Faubourg Saint Jacques, 75014 Paris, France; 2 Université Paris Descartes, Sorbonne Paris Cité, Faculté de médecine, 15 rue de l'Ecole de Médecine, 75006 Paris, France; 3 Cochin Institute, INSERM U1016/CNRS UMR8104, 22 rue Méchain, 75014 Paris, France; 4 Intensive Care Unit, Versailles Hospital, 177 rue de Versailles, 78150 Le Chesnay, France

## Abstract

**Introduction:**

Community-acquired pneumonia (CAP) account for a high proportion of ICU admissions, with *Streptococcus pneumoniae *being the main pathogen responsible for these infections. However, little is known on the clinical features and outcomes of ICU patients with pneumococcal pneumonia. The aims of this study were to provide epidemiological data and to determine risk factors of mortality in patients admitted to ICU for severe *S. pneumoniae *CAP.

**Methods:**

We performed a retrospective review of two prospectively-acquired multicentre ICU databases (2001-2008). Patients admitted for management of severe pneumococcal CAP were enrolled if they met the 2001 American Thoracic Society criteria for severe pneumonia, had life-threatening organ failure and had a positive microbiological sample for *S. pneumoniae*. Patients with bronchitis, aspiration pneumonia or with non-pulmonary pneumococcal infections were excluded.

**Results:**

Two hundred and twenty two patients were included, with a median SAPS II score reaching 47 [36-64]. Acute respiratory failure (n = 154) and septic shock (n = 54) were their most frequent causes of ICU admission. Septic shock occurred in 170 patients (77%) and mechanical ventilation was required in 186 patients (84%); renal replacement therapy was initiated in 70 patients (32%). Bacteraemia was diagnosed in 101 patients. The prevalence of *S. pneumoniae *strains with decreased susceptibility to penicillin was 39.7%. Although antibiotherapy was adequate in 92.3% of cases, hospital mortality reached 28.8%. In multivariate analysis, independent risk factors for mortality were age (OR 1.05 (95% CI: 1.02-1.08)), male sex (OR 2.83 (95% CI: 1.16-6.91)) and renal replacement therapy (OR 3.78 (95% CI: 1.71-8.36)). Co-morbidities, macrolide administration, concomitant bacteremia or penicillin susceptibility did not influence outcome.

**Conclusions:**

In ICU, mortality of pneumococcal CAP remains high despite adequate antimicrobial treatment. Baseline demographic data and renal replacement therapy have a major impact on adverse outcome.

## Introduction

Community-acquired pneumonia (CAP) is a frequent and severe infection, and is considered the primary cause of death from infection, and the sixth most common cause of overall mortality in Western countries [1,2]. Consequently, CAP represents one of the leading causes of infectious admissions to the intensive care unit (ICU) [3]. Indeed, the latest studies have reported that up to 10 % of all patients hospitalised with CAP require ICU management [4]. In this specific subgroup of severely ill patients, the overall mortality rate remains unacceptably high despite improvement in critical care management [5]. Furthermore, the medical burden of CAP is very high in terms of direct costs, associated morbidity and long-term disability [6,7].

*Streptococcus pneumoniae *(*S. pneumoniae*) is the principal causative agent of CAP requiring hospital or ICU admission [8]. Paediatric and adult literature about non-severe pneumococcal pneumonia is abundant, but specific data on patients requiring ICU admission are scarce. In the two studies focusing on the epidemiology of pneumococcal pneumonia among patients admitted to ICU, co-morbidities negatively influenced patient outcomes, but were over-weighted by the severity of the clinical features [9,10]. Recent therapeutic researchers have pointed out that early and adequate antibiotherapy is of greatest importance during sepsis, whereas adjuvant therapies like steroids or activated protein C may reduce the fatality rate [11]. However, the roles of these anti-inflammatory agents, as well as the association with macrolides during severe pneumococcal pneumonia are a matter of debate [12]. Thus, increased knowledge of severe *S. pneumoniae *pneumonia that may improve early detection and treatment of this particular subgroup carries high interest for ICU physicians.

The aim of this present study was to provide recent epidemiological data through a large cohort of adult patients admitted to ICU for severe pneumococcal CAP. In addition to analysis of microbiological features, we assessed the respective influence of co-morbidity and organ failure on mortality. We also investigated the potential impact of adjuvant therapies on outcome.

## Methods and materials

### Study design

After approval from the local Cochin Hospital institutional review board, patients were retrospectively selected from two prospective cohorts including ICU patients admitted with infection (one multicentre cohort and one from the Cochin medical ICU) between January 2001 and June 2008. Informed consent was waived and informed assessment was obtained from all patients or next of kin before inclusion.

### Inclusion and exclusion criteria

Inclusion criteria were 1) age over 18 years; 2) severe CAP diagnosed according to the adapted American Thoracic Society definition [13,14], which includes features consistent with pneumonia (new or increased cough with or without sputum production, tachypnoea, chest pain, abnormal temperature (> 38°C or < 36°C) or lung consolidation on physical examination), with either one of two major criteria (need for mechanical non-invasive or invasive ventilation or septic shock) or any two of three minor criteria (involvement of more than two lobes on a chest radiograph, systolic blood pressure < 90 mmHg or PaO_2_/FIO_2 _ratio < 250 mmHg); 3) ICU hospitalisation required for haemodynamic, respiratory or neurologic failure or severe co-morbidities and 4) a microbiological sample positive for *S. pneumonia*, that is, sputum examination with a bacterial count ≥ 10^7 ^colony forming unit/mL (CFU/mL) (fulfilling the usual quality criteria of > 25 polymorphonuclear leukocytes and < 10 epithelial cells per low-power field, magnification × 100), or a microbiological sample positive for *S. pneumoniae *in a normally sterile site (a positive quantitative culture of endotracheal aspirate, with a bacterial count ≥ 10^6 ^CFU/mL, a positive quantitative culture of broncho-alveolar lavage with a bacterial count ≥ 10^4 ^CFU/mL, or a protected specimen brush with a bacterial count ≥ 10^3 ^CFU/mL, or a positive blood culture, or a positive pleural culture or a positive urinary antigen).

Healthcare-associated pneumonia was not included. Patients with bronchitis or aspiration pneumonia due to *S. pneumoniae *and patients with concomitant pneumococcal meningitis or endocarditis were excluded from the study.

### Study variables and outcomes

Data were prospectively collected, including demographic characteristics, initial clinical presentation, usual biological values, antibiotherapy management, organ failures and outcomes were investigated. Septic shock was defined as the requirement for vasopressor for more than 4 hours or hypotension (systolic blood pressure < 90 mmHg) for more than one hour despite adequate fluid challenge plus either a change in mental status, oliguria, organ dysfunction, or lactate > 2 mmol/L [15]. Acute respiratory distress syndrome (ARDS) and multi-organ failure were defined according to the usual criteria [15,16]. Acute kidney injury was considered present if the patient was considered to be in the Injury stage of the Risk, Injury, Failure, Loss and End-stage disease (RIFLE) criteria [17]. The Logistic Organ Dysfunction System (LODS) score [18] and the Simplified Acute Physiology Score II (SAPS II) [19] were also calculated. The following therapeutic data were recorded: vasoactive drugs, renal replacement therapy, activated protein C and low doses of systemic corticosteroids.

All patients were followed up until death or hospital discharge. Two independent investigators (NM and AM) reviewed all files.

Susceptibility of the bacterial strains to penicillin G was recorded according to the recommendations from the Antibiogram Committee of the French Society for Microbiology (CA-SFM) [20] for antimicrobial susceptibility testing and breakpoints and defined as susceptible: penicillin minimal inhibitory concentration (MIC) ≤ 0.06 mg/L, or intermediate: 0.06 mg/L < MIC ≤ 1 mg/L; resistant: MIC > 1 mg/L.

Antibiotherapy was considered appropriate if the initial empiric therapy had *in vitro *activity against the isolated strain of *S. pneumoniae*. Pneumococcal vaccine status was documented for less than 5% of the records and thus was not included in the analysis.

### Statistical analysis

Continuous variables were expressed as median and interquartile range, and categorical variables as number and percentage. Continuous variables were compared using the Mann-Whitney *U*-test and categorical variables were compared using the chi-square test. Independent predictors of outcome were assessed using a multivariate logistic regression model through a stepwise forward procedure, where the variable of interest was hospital mortality. Results were expressed as odds ratio (OR) and 95% confidence interval (CI). For continuous variables, the OR was indexed to an increment of one unit. Categorical variables were expressed as absence or presence. Goodness-of-fit of the final model was assessed by the Hosmer-Lemeshow test. Statistical significance was defined as *P *< 0.05. Analyses were performed using PASW software (SPSS Inc., Chicago, ILL, USA).

## Results

Of the 3,658 patients enrolled in the two cohorts, 423 had positive microbiological samples for *S. pneumoniae*. Among them, 133 had evidence of aspiration or bronchitis and 290 were subsequently screened. After the exclusion of 68 patients with concomitant meningitis, 222 patients were finally included in this study.

### Patients' characteristics

**Figure 1:**
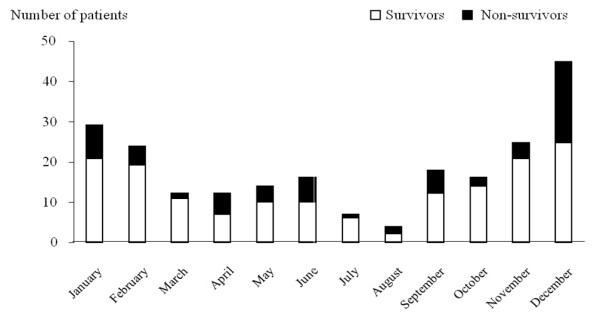
**Seasonal variation in ICU admission for severe pneumococcal community-acquired pneumonia**.

Demographic characteristics and the main clinical features of patients are reported in Table 1. Median (range) SAPS II and LODS scores were respectively 47 (36 to 64) and 8 (5 to 10), thereby reflecting severe critical illness. Eighty-four patients had no tobacco or alcohol intoxication, and no coexisting illness. Twenty-three patients had already experienced an invasive pneumococcal infection in the past. Repartition of the disease onset according to the season is represented in Figure 1, with an incidence peak during autumn and winter.

**Table 1:** Baseline demographics and clinical characteristics of patients

	All patients n = 222	Survivors n = 158	Non-survivors n = 64	*P*
**Demographic**				
Age	60 (49-75)	59 (48-70)	67 (54-80)	0.003
Male gender, n (%)	146 (66)	96 (57)	50 (78)	0.014

**Underlying conditions**				
Tobacco use, n (%)	89 (40)	62 (37)	27 (42)	0.5
Alcohol abuse, n (%)	65 (29)	44 (26)	21 (33)	0.34
COPD, n (%)	57 (26)	36 (21)	21 (33)	0.08
Immunosuppressive treatment, n (%)	49 (22)	32 (19)	17 (26)	0.36
Cirrhosis, n (%)	14 (6)	5 (3)	9 (14)	0.002
Diabetes mellitus, n (%)	32 (14)	21 (12)	11 (17)	0.43
Chronic heart failure, n (%)	23 (10)	10 (6)	13 (20)	0.002
Chronic renal insufficiency, n (%)	12 (5)	6 (3)	6 (9)	0.1
Asplenia, n (%)	3 (1)	2 (1)	1 (2)	0.86
Systemic disease, n (%)	11 (5)	7 (4)	4 (6)	0.56
Cancer, n (%)	23 (10)	12 (7)	11 (17)	0.03
Long-term steroid therapy, n (%)	29 (13)	20 (12)	9 (14)	0.68

**Clinical feature**				
SAPS II	47 (36-64)	43 (34-56)	63 (49 75)	< 0.001
Septic shock, n (%)	170 (76)	107 (64)	63 (98)	< 0.001
ARDS, n (%)	100 (45)	57 (34)	43 (67)	< 0.001
Ventilator-acquired pneumonia, n(%)	69 (31)	50 (32)	19 (30)	0.87
Bacteraemia, n (%)	101 (45)	73 (43)	28 (31)	0.71
Penicillin-susceptible *S. pneumoniae *strain, n (%) (over 194 patients)	117 (60)	83 (53)	34 (53)	0.99

**Main laboratory findings on admission**				
PaO_2_/FiO_2 _ratio	98 (73-149)	100 (75-156)	91 (61-128)	0.12
Lactate (mmol/L)	2.9 (1.6-5.9)	2.2 (1.3-5.6)	5.16 (2.6-9.1)	0.02
Glycaemia (mmol/L)	8.3 (5-13)	8.3 (4.9-12.6)	7.3 (4.9-11.2)	0.7
Urea (mmol/L)	10.7 (6.8-16.7)	9.3 (6.3-14.5)	12 (9.3-20.3)	0.13
Platelet count < 10,0000/mm^3^, n (%)	52 (23)	32 (19)	20 (31)	0.05
Leukocytes < 1000/mm^3^, n (%)	56 (25)	32 (19)	22 (34)	0.05
Prothrombin time < 50%, n (%)	55 (25)	30 (18)	25 (39)	< 0.001
Bilirubin > 25 mmol/L, n (%)	59 (27)	26 (15)	23 (36)	0.001

**Treatments**				
Mechanical ventilation, n (%)	186 (84)	123 (73)	63 (98)	< 0.001
Renal replacement therapy, n (%)	70 (32)	30 (18)	40 (62)	< 0.001
Antibiotherapy combination including macrolides, n (%)	163 (73)	110 (65)	53 (83)	0.04
Low-dose steroids, n (%)	69 (31)	41 (24)	28 (31)	0.001
Activated protein C, n (%)	44 (20)	32 (19)	12 (19)	0.78

Data are expressed as median and interquartile range. COPD: chronic obstructive pulmonary disease; SAPS: Simplified Acute Physiology Score; ARDS: acute respiratory distress syndrome; *S.pneumoniae*: *Streptococcus pneumonia*.

### Clinical features

Reasons for ICU admission were mainly acute respiratory failure (n = 154), septic shock (n = 54), coma (n = 6) or others (n = 8). Twenty-eight patients were admitted after worsening of their clinical status in the ward. On admission, most patients had several organ dysfunctions with a median organ failure score of 3 (range 2 to 4). During the ICU stay, 157 patients (70.7%) had multi-organ failure. Respiratory function was severely impaired with a median PaO_2_/FiO_2 _ratio of 98 (73 to 149) at admission, and multilobar pneumonia was present in 68 patients (30.6%). Non-invasive ventilation was initiated in 70 patients but 52 patients required secondary endotracheal intubation. Overall, invasive ventilation was required for 186 patients, of whom 100 (45%) exhibited ARDS criteria. In survivors, ventilator weaning was achieved after a median of 8 (4 to 20) days.

In addition to severe hypoxemia, more than 50% of the patients had elevated plasma lactate concentrations and one fourth of the patients presented other biological severity signs such as leucopoenia or disseminated intravascular coagulation criteria (Table 1).

During the course of pneumonia, 170 patients (76.6%) developed septic shock, catecholamine infusion was required for a median of 3 (0 to 7) days. At admission, acute kidney injury was present in 87 patients and renal replacement therapy was initiated in 70 patients (31.5%). Median mechanical ventilation-free, catecholamine-free and renal replacement therapy-free days were respectively 3 (1 to 6), 7.5 (3 to 16) and 12 (4 to 19) days. Adjuvant therapies, including low dose steroids or activated protein C, had been initiated in 69 (31%) and 44 (20%) patients respectively.

### Microbiological data and antibiotherapy

*S. pneumoniae *CAP was documented by direct pulmonary microbiological sampling in 150 (68%) cases (tracheobronchial aspirates, n = 76; blind protected telescoping catheter, n = 40; sputum examination, n = 24; broncho-alveolar lavage, n = 10). Pleural effusion was also positive in 12 patients and urinary antigen in 58 patients. In 28 patients, the latter examination was the only diagnostic method with a positive result. As commonly reported in pneumococcal pneumonia, positive blood culture was found in 101 patients (45.5%), and was the single microbiological proof in 33 patients. It is noteworthy that patients with or without bacteraemia had similar organ failure features (Table 2). Co-infection with other bacteria was demonstrated in just 12 patients. Nosocomial infections occurred in 69 patients during the ICU stay.

**Table 2:** Comparison of patients with or without pneumococcal bacteraemia

	Patient with bacteraemia n = 101	Patient without bacteraemia n = 121	*P*
Septic shock	76 (76)	94 (78)	0.69
Mechanical ventilation	83 (83)	103 (85)	0.46
ARDS	51 (51)	49 (40)	0.17
Acute kidney injury	36 (36)	51 (42)	0.33
Need for renal replacement therapy	35 (35)	35 (29)	0.46
	72 (72)	85 (70)	0.83
Multi-organ failure	26 (26)	33 (28)	0.77
ICU mortality Hospital mortality	28 (28)	36(28)	0.71

Data are expressed as n (%). ARDS: acute respiratory distress syndrome.

The prevalence of *S. pneumoniae *strains with decreased susceptibility to penicillin was 39.7% (n = 77), and resistance rates for macrolides and fluoroquinolones were 54% and 1% respectively. Initial antibiotherapy was adequate against *S. pneumoniae *in 92.3% (179 patients out of 194, after exclusion of patients with pneumococcal CAP diagnosed simply with positive urinary antigen). As recommended by international guidelines for severe CAP treatment, antimicrobial therapy including macrolides was initiated in a large proportion of the population (163 patients, 73.4%).

### Outcome and prognostic factors

The median ICU length of stay was 13 (6 to 25) days. The ICU mortality rate was 26.6% (n = 59 patients), including 15 patients who died within the first five days. The overall hospital mortality rate was 28.8% (n = 64 patients). Causes of ICU mortality were multi-organ failure (n = 38), refractory hypoxemia (n = 10), withdrawal or withholding of life support (n = 7) and persistent neurological failure (n = 4). ICU mortality was not significantly lower in early ICU admissions (22.6% of ICU referrals within 12 hours of onset of infectious or respiratory symptoms vs. 27.5% of admissions after a delay of more than 12 hours, *P *= 0.41). Interestingly, ICU mortality was 31.1% when at least one of the two major criteria of the American Thoracic Society was present (n = 190), whereas all patients with at least two of the three minor ATS/IDSA criteria survived (n = 32).

In univariate analysis, clinical variables associated with increased mortality included age; male gender; co-morbidities, such as cirrhosis, chronic heart failure and cancer; the SAPS II; organ dysfunctions such as ARDS and septic shock; organ support, such as mechanical ventilation and renal replacement therapy; biological disorders (lactate, coagulation disorders) and adjunctive therapies (low dose corticosteroids and association of antibiotics) (Table 1). In order to identify independent prognostic factors, we performed a multivariate analysis that included significant variables from the univariate analysis. Because of its co-linearity with several variables, the SAPS II was not included in the final model. Age, male sex and renal replacement therapy were identified as independent predictors of hospital mortality (Table 3). In contrast, shock (OR 12.1, 95% CI 0.78, 187.19), acute kidney injury (OR 1.01, 95% CI 0.48, 2.17), thrombocytopenia (OR 1.74, 95% CI 0.54, 5.66), leukopenia (OR 1.03, 95% CI 0.39, 2.69) and low-dose steroids use (OR 1.34, 95% CI 0.61, 2.96]) were not significantly associated with hospital mortality.

**Table 3:** Multivariate analysis of factors associated with hospital mortality

	Odds ratio (95% CI)	*P*
Male sex	2.83 (1.16, 6.91)	0.01
Age	1.05 (1.02, 1.08)	0.026
Renal replacement therapy	3.78 (1.71, 8.36)	0.001

## Discussion

We report here the most important cohort of pneumococcal pneumonia requiring ICU management. The strength of our study is that we studied a homogenous population, including patients with only microbiologically documented *S. pneumoniae *CAP, with exclusive pulmonary infection and reviewing of the patients' files by two independent investigators. Moreover, we chose to eliminate aspiration pneumonia or chronic obstructive pulmonary disease (COPD) exacerbated by *S. pneumoniae *colonisation. We used a multicentre cohort, reflecting both university (tertiary referral) and non-university hospitals with a wide scope of hospital sizes, locations and practice environments.

It is striking that underlying co-morbidities have no impact on outcome, even if some of them have been demonstrated to increase the incidence of invasive pneumococcal disease [21]. If tobacco and alcohol abuse were more frequent in the pneumococcal CAP than in the general population, they did not influence the course of the infection. Similarly, respiratory failure and septic shock were the main admission motives, but they were not independent prognostic variables. In contrast, male gender, age and need for renal replacement therapy were overwhelmingly independent risk factors for mortality. In a set of 43 patients with severe pneumococcal pneumonia, death was driven by organ failure rather than co-morbidity [10]. In another group of 137 ICU patients, Georges *et al*. showed similar results, except for renal replacement therapy [9]. These authors reported that leucopenia, nosocomial infections and inadequacy of initial antimicrobial therapy were other risk factors for death. In our study, none of these factors were associated with adverse outcomes. Astonishingly, male gender had a major impact on outcome. This is in line with the higher mortality rate of sepsis in male patients despite lower incidence than in female patients [22]. Underlying hormonal-, social- or disease-associated mechanisms remain to be explored to explain these findings [23].

Pneumococcal pneumonia requiring ICU admission is associated with a high rate of fatality, with more than one fourth of the patients dying in hospital. The highest proportion of death occurs early in the course of the disease, despite an excellent proportion of initial adequate antibiotherapy [24]. This is considered to be related to the early inflammatory process, which is overwhelming host defences. In a recent study, Garcia-Vidal *et al*. investigated independent factors associated with early deaths in CAP and demonstrated that age, altered mental status, multilobar pneumonia, shock, bacteraemia and inadequate empiric antibiotic therapy were predictors of death within 48 hours [25]. The small number of patient dying in the first two days in our cohort did not allow us to perform similar analysis.

The present study confirmed that *S. pneumoniae *pneumonia-related bacteraemia is not associated with a worse outcome. Clinical features and organ failures prevent identification of bacteraemic and non-bacteraemic patients (Table 2). This observation is similar to previous reports [26,27] including the study from Bordon *et al*. who investigated a cohort of 1,847 patients, including 125 patients with bacteraemia, and found that prognosis was not influenced by *S. pneumoniae *bloodstream infection [27]. Our findings are in line with prior investigations, suggesting that presence of pneumococcal bacteraemia might not be a contraindication for de-escalation and short duration of antibiotherapy in clinically stable patients.

As recommended by international guidelines [28], initial antibiotherapy in the setting of severe CAP must include a *β*-lactamin active against *S. pneumoniae*. As a result, the rate of adequate antibiotherapy is usually high, as reported in our study. Consequently, improvement of antibiotherapy delivery is unlikely to improve the outcome of pneumococcal pneumonia. Thus, adjuvant therapies might have a determinant role to reduce mortality rate [9,24,29]. In our study, we failed to demonstrate that use of activated protein C was associated with a better outcome, despite the suggestion by Laterre *et al*. that its use was of particular interest during *S. pneumoniae *pneumonia in the Prowess study [30]. However, the recent Prowess Shock study did not replicate these results and led to drug retrieval [31]. Similarly, the Captivate study was the largest clinical trial conducted on severe CAP and examined the recombinant tissue factor pathway inhibitor. With 694 proven cases of pneumococcal pneumonia, the study failed to demonstrate any benefit on survival in either the whole population or the pneumococcal subgroup [32].

Low-dose steroids represent another controversial therapy in severe sepsis. Some studies suggest this treatment improves survival in critically ill patients with severe CAP [33]. In contrast, others, such as Snijders *et al.*, who compared the administration of corticosteroids plus antibiotics, vs. antibiotics alone in severe sepsis, reported no benefit with steroids [34]. The low number of treated patients, the observational characteristics of our study and the variability of steroid use between the participating centres preclude drawing any conclusions. However, in the present study, low doses of steroids were associated with increased mortality in univariate analysis. With the findings of the Corticus study, low-dose steroids should be restricted to patients with refractory septic shock [35].

Association of *β*-lactamin and either a macrolide or a fluoroquinolone has been linked with a lower mortality in severe CAP [8,36]. However, the clinical impact of such a combination is seriously debated, and may vary among pathogens. For *S. pneumoniae*, combined antibiotic therapy might be beneficial to treat undiagnosed co-infections, or through anti-inflammatory properties of macrolides. However, these data emerged essentially from retrospective or small prospective studies [8,36-42], and are challenged by several other reports [43-48] as with our study, and will have to be confirmed in a multicentre randomized study. To date, the recommendation of dual antibiotherapy for *S. pneumoniae *CAP has not been endorsed by scientific societies.

Pneumococcal pneumonia occurred preferentially during autumn or winter. This phenomenon has already been described in non-critically ill patients, with a clear link to external temperature [49] and concomitant respiratory viral infections [50]. Here we report for the first time this seasonality in ICU pneumococcal CAP. Interestingly, the severity of pneumonia assessed by the mortality rate was not influenced by season.

We found that *S. pneumoniae *strains with diminished susceptibility to penicillin were involved in almost 40% of cases. This is consistent with the recent report from the French national reference centre for *S. pneumoniae*, which collects isolates from invasive pneumococcal diseases [51]: the overall proportion of strains that are non-susceptible to penicillin decreased from 45% to 35% in adults between 2001 and 2006. Interestingly, we confirmed in this severe ICU population that penicillin resistance did not influence outcome, as previously described in less severe populations [52,53].

A few limitations deserve careful consideration. First, despite prospective data acquisition, there were some missing data, as illustrated by the lack of information about influenza or pneumococcal vaccination or HIV status. Moreover, we did not have access to the time lag between symptom onset and antibiotherapy initiation, which has been proposed as a major outcome determinant [54]. Second, due to the multicentre design, therapeutic strategies (that is, antimicrobial therapy, ventilation protocols and adjunctive therapies) were not standardised. Third, we used the 2001 American Thoracic Society guidelines for severe CAP definition, which included three minor criteria, rather than the 2007 ATS/IDSA definition that includes nine minor criteria [28]. Phua *et al*. investigated the prognostic value of minor criteria for ICU admission, according to the 2007 guidelines, and found that mortality rose from 0.9% with no risk factors to 35.2% for patients with at least three minor criteria [55]. This underlines the large improvement of determination of prognosis provided with the 2007 criteria, as compared with the initial one that we used. Unfortunately, several of the new minor criteria were not present in our databases. Fourth, we could not study the serotypes of *S. pneumoniae *in our analysis, and strain specificities seem to be a major element of severity [56]. This requires specific investigations, which were not routinely performed. Similarly, biomarkers such as C-reactive protein or procalcitonin were not available in the databases, whereas routine use of these biomarkers has been shown to improve the triage score for CAP patients in the emergency department [57]. As all our patients were hospitalised in the ICU, the interest of such predictor tools remains to be demonstrated in this population.

## Conclusions

In summary, we conducted a multicentre observational study of the epidemiology and outcome of pneumococcal CAP in a large homogeneous cohort of critically ill patients. We highlighted that *S. pneumoniae *pneumonia was still associated with a poor outcome, with high early mortality, despite adequate antimicrobial therapy. In the future, determination of factors related to the host, that is, genetic susceptibility, and to the bacteria, that is, virulence, is likely to be crucial for a better understanding of the pathophysiology of invasive pneumococcal pneumonia [58].

## Key messages

• Despite a high proportion of adequate antibiotherapy, mortality in patients with severe pneumococcal CAP admitted to the ICU reaches 29 %.

• Microbiological specificities (penicillin susceptibility and concomitant bacteraemia) have no significant impact on organ failure and mortality.

• Underlying co-morbidities do not impair survival in the ICU, whereas age, male sex and need for renal replacement therapy are associated with an unfavourable outcome.

• Adjunctive therapies do not seem to influence the impact of severe pneumococcal pneumonia.

## Abbreviations

ARDS: acute respiratory distress syndrome; CFU: colony forming unit; COPD: chronic obstructive pulmonary disease; HIV: human immunodeficiency virus; ICU: Intensive Care Unit; LODS: Logistic Organ Dysfunction System; MIC: minimal inhibitory concentration; NYHA: New York Heart Association; SAPS: Simplified Acute Physiology Score; *S.pneumoniae*: *Streptococcus pneumonia*.

## Competing interests

JC, AC and JPM are consultants for Lilly. The authors declare that they have no other competing interests relevant to the field of the manuscript.

## Authors' contributions

NM, AM, JPB and JPM designed the study. NM, AM and AB extracted the data. FP performed the statistical analysis. NM, AM, AB, FP, VL, JC, AC, JDC, JPB and JPM contributed to the conduct of study and data analysis. NM and JPM wrote the manuscript. All the authors read and approved the final version of the manuscript.
